# Interleukin-6 and Cyclooxygenase-2 downregulation by fatty-acid fractions of *Ranunculus constantinopolitanus*

**DOI:** 10.1186/1472-6882-9-44

**Published:** 2009-11-16

**Authors:** Sabreen F Fostok, Rima A Ezzeddine, Fadia R Homaidan, Jamal A Al-Saghir, Ralph G Salloum, Najat A Saliba, Rabih S Talhouk

**Affiliations:** 1 Department of Biology, Faculty of Arts and Sciences, American University of Beirut, Beirut, Lebanon; 2 Department of Chemistry, Faculty of Arts and Sciences, American University of Beirut, Beirut, Lebanon; 3 Department of Physiology, Faculty of Medicine, American University of Beirut, Beirut, Lebanon; 4 Nature Conservation Center for Sustainable Futures (IBSAR), American University of Beirut, Beirut, Lebanon; 5 Department of Internal Medicine/Pediatrics, School of Medicine, Wayne State University, Detroit, MI 48201, USA

## Abstract

**Background:**

Medicinal plants represent alternative means for the treatment of several chronic diseases, including inflammation. The genus *Ranunculus*, a representative of the Ranunculaceae family, has been reported to possess anti-inflammatory, analgesic, antiviral, antibacterial, antiparasitic and antifungal activities, possibly due to the presence of anemonin and other. Different studies have shown the occurrence of unusual fatty acids (FAs) in Ranunculaceae; however, their therapeutic role has not been investigated. The purpose of this study is to characterize potential anti-inflammatory bioactivities in *Ranunculus constantinopolitanus *D'Urv., traditionally used in Eastern Mediterranean folk medicine.

**Methods:**

The aerial part of *R. constantinopolitanus *was subjected to methanol (MeOH) extraction and solvent fractionation. The bioactive fraction (I.2) was further fractionated using column chromatography, and the biologically active subfraction (Y_2+3_) was identified using infrared (IR) spectroscopy, nuclear magnetic resonance (NMR) and gas chromatography-mass spectrometry (GC-MS). The effects of I.2 and Y_2+3 _on cell viability were studied in mouse mammary epithelial SCp2 cells using trypan blue exclusion method. To study the anti-inflammatory activities of I.2 and Y_2+3_, their ability to reduce interleukin (IL)-6 levels was assessed in endotoxin (ET)-stimulated SCp2 cells using enzyme-linked immunosorbent assay (ELISA). In addition, the ability of Y_2+3 _to reduce cyclooxygenase (COX)-2 expression was studied in IL-1-treated mouse intestinal epithelial Mode-K cells via western blotting. Data were analyzed by one-way analysis of variance (ANOVA), Student-Newman-Keuls (SNK), Tukey HSD, two-sample t-test and Dunnett t-tests for multiple comparisons.

**Results:**

The chloroform fraction (I.2) derived from crude MeOH extract of the plant, in addition to Y_2+3_, a FA mix isolated from this fraction and containing palmitic acid, C18:2 and C18:1 isomers and stearic acid (1:5:8:1 ratio), reduced ET-induced IL-6 levels in SCp2 cells without affecting cell viability or morphology. When compared to fish oil, conjugated linoleic acid (CLA) and to individual FAs as palmitic, linoleic, oleic and stearic acid or to a mix of these FAs (1:5:8:1 ratio), Y_2+3 _exhibited higher potency in reducing ET-induced IL-6 levels within a shorter period of time. Y_2+3_ also reduced COX-2 expression in IL-1-treated Mode-K cells.

**Conclusion:**

Our studies demonstrate the existence of potential anti-inflammatory bioactivities in *R. constantinopolitanus *and attribute them to a FA mix in this plant.

## Background

Dietary supplements are used as preventive means to maintain a healthy state. Among them, polyunsaturated fatty acids (PUFAs), specifically members of the omega (*n*)-3 series and conjugated linoleic acid (CLA), have become the focus of extensive nutritional research in the last decade [1-6] due to their reported anti-inflammatory properties. Indeed, CLA and the *n*-3 eicosapentaenoic acid (EPA) and docosahexaenoic acid (DHA) were found to reduce the levels of many inflammatory mediators, including cytokines: interleukin (IL)-1, IL-6 and tumor necrosis factor (TNF)-α, eicosanoids: prostaglandins (PGs), thromboxanes (TXs) and leukotrienes (LTs), enzymes: cyclooxygenase (COX)-2 and 5-lipoxygenase (LOX), adhesion molecules: E-selectin, intercellular adhesion molecule (ICAM)-1 and vascular cell adhesion molecule (VCAM)-1 and matrix metalloproteinases (MMPs) [7-10]. Clinically, reports have suggested that supplementation with *n*-3 fatty acids (FAs) has beneficial effects in chronic inflammatory diseases such as inflammatory bowel disease (IBD), rheumatoid arthritis (RA) and psoriasis [11]. Moreover, adding *n*-3 FAs to the diet of patients with hypertriglyceridemia is now recognized as an efficient triglyceride-lowering therapeutic measure [12]. CLA has been shown to possess similar effects, though the safety and efficacy of CLA dietary supplements is still under investigation [9,13,14].

In contrast to the numerous reports investigating the protective effects of animal-derived PUFAs in inflammation, the literature describing anti-inflammatory bioactivities in plant-derived FAs, and particularly those existing in the Ranunculaceae family, used as a medicinal herb, is poor. This family, comprising around 2500 species distributed all over the world, is especially widespread in slow streams, ditches and shallow ponds of muddy mineral-rich water. *Ranunculus*, a representative genus of the Ranunculaceae family, has been established as an anti-inflammatory, analgesic, antiviral, antibacterial, antiparasitic and antifungal agent [15-20]. Such properties of this genus could be due to the presence of anemonin, a dimerization product of the γ-lactone protoanemonin, both of which have been shown to possess several pharmacological effects [18,21-23]. In this paper, the effect of a FA mix extracted from *Ranunculus constantinopolitanus *D'Urv., an Eastern Mediterranean plant that extends as far as Armenia, on endotoxin (ET)-induced IL-6 and IL-1-induced COX-2 in SCp2 and Mode-K cells, respectively, is investigated. This effect is subsequently compared to the activities of over-the-counter products reported to modulate inflammation, including fish oil and CLA.

## Methods

### Cell culture

Mouse mammary epithelial cell strain, SCp2 cells (kindly provided as a gift by P.Y. Desprez, Geraldine Brush Cancer Research Institute, California Pacific Medical Center, San Francisco, CA, USA) were cultured as previously described by Saliba *et al*. (2009) [24]. Briefly, cells were grown on 100-mm tissue culture plates (BD Falcon, Franklin Lakes, NJ, USA) in growth medium (GM) consisting of Dulbecco's Modified Eagle's Medium Nutrient Mixture/F12 Ham (DMEM/F12; Gibco, Paisley, Scotland) supplemented with 5% heat-inactivated fetal bovine serum (FBS; Gibco), insulin (5 μg/ml; Sigma, St. Louis, MO, USA) and 1% penicillin/streptomycin mixture (Gibco) in a humidified incubator (95% air 5% CO_2_; VWR Scientific, West Chester, PA, USA) at 37°C. Upon confluency, cells were detached by trypsinization and replated in GM either on 100-mm tissue culture plates for maintenance or on 6-well plates (BD Falcon) at 1 × 10^6 ^cells/well to be used in different experiments. On the second day after plating, cells were shifted to differentiation medium (DM) consisting of DMEM/F12 supplemented with insulin (5 μg/ml), hydrocortisone (1 μg/ml; Sigma), ovine prolactin (3 μg/ml; Sigma) and 1% penicillin/streptomycin. On the third day, unless otherwise indicated, plant and ET treatments were performed in triplicates.

Murine intestinal epithelial cell type Mode-K cells were cultured as previously described by Homaidan *et al*. (2003) [25]. Briefly, cells were maintained in DMEM containing 1 g/L glucose and 10 mM sodium pyruvate (Invitrogen, Carlsbad, CA, USA) and supplemented with 10% FBS (Invitrogen), 1% non-essential amino acids (Invitrogen) and 0.5% penicillin/streptomycin (Invitrogen). At 70-80% confluency, cells were detached by trypsinization and replated on tissue culture flasks (BD Falcon) for maintenance or on 6-well plates at 2 × 10^5 ^cells/well to be used in different experiments. On the second day after plating, cells were starved. Plant and IL-1 treatments were performed on the second and third days.

### Extraction of plant material

#### Collection and drying

*Ranunculus constantinopolitanus *was collected from Yanta, Lebanon, located at an altitude of 1395 m. A voucher specimen of the plant (voucher number: 72) was deposited at the Post Herbarium of the American University of Beirut, Beirut, Lebanon. Following collection, the aerial part (stems, leaves and flowers) of *R. constantinopolitanus *was dried by leaving the plant sample in the shade for two weeks before grinding it into approximately 10-mm pieces using a blender. Ground samples were subjected to solvent extraction using methanol (MeOH) or else they were stored at -20°C for later use.

#### Methanol extraction and fractionation

The crude MeOH extract was subjected to further solvent fractionation as previously described by Saliba *et al*. (2009) [24]. In brief, the dried plant material was subjected to extraction through soaking in MeOH (1:10 w/v) for 16 hr. Incubation on a shaker at 20°C occurred in the first 2 hr, and then the plant sample was left in MeOH for the remaining time. The MeOH extract was filtered using a cheese cloth (sterile gauze sponges 30 × 30 cm) to give a solid phase (R-I) and a filtrate numbered "I" and referred to as "crude MeOH extract". R-I was soaked in ethyl acetate (EtOAc) at a ratio of 10:1 (w/v). It was then separated by filtration into a solid phase and a filtrate numbered "I.1". The crude MeOH extract (I) was evaporated to 1/10 of its volume at less than 40°C, acidified to pH 2 by concentrated H_2_SO_4 _and then separated into an aqueous and an organic layer using a mixture of CHCl_3 _and H_2_O (2:1 v/v). The organic layer was collected and labelled as "I.2". The aqueous layer was basified to pH 10 by the addition of concentrated NH_4_OH and was then resuspended in a mixture of CHCl_3 _and MeOH (3:1 v/v) (the total volume of CHCl_3_:MeOH is equal to four times the volume of the aqueous layer) to be later separated into an organic layer and an aqueous layer labelled as "I.3" and "I.4", respectively. I.1, I.2, I.3 and I.4 were evaporated to dryness under vacuum, weighed, dissolved in 100% ethanol (EtOH) and stored in dark bottles at -20°C. The fractions were subsequently screened for potential biological activities, and the active ones were selected for further purification.

#### Separation and identification of subfraction Y_2+3_

Biologically active fraction I.2 (6 g) was re-dissolved in a minimum volume of petroleum ether (P.ether):CHCl_3_:EtOAc mixture (2:2:1) and was applied to a column chromatography consisting of 800 g of silica gel. A gradient elution was performed using P.ether:CHCl_3_:EtOAc (2:2:1) (6500 ml), followed by P.ether:CHCl_3_:EtOAc (1:3:1) (4000 ml), CHCl_3_:EtOAc:MeOH (3:3:1) (4200 ml) and MeOH, successively. Subfraction Y_2+3 _was collected using P.ether:CHCl_3_:EtOAc (2:2:1) as a mobile phase.

Y_2+3_, which had the highest biological activity, was purified via solid phase extraction (SPE). Spectroscopic data using infrared (IR) spectroscopy and nuclear magnetic resonance (NMR) showed that Y_2+3 _was a mixture of FAs. The mixture was consequently converted to FA methyl esters (FAMEs) and resolved into individual components using gas chromatography-mass spectrometry (GC-MS). GC analysis was performed using a Trace™ gas chromatograph equipped with HP-5 capillary column (30 m long, 250 μm i.d and 0.25 μm film thickness), Helium as a carrier at a flow rate of 1 ml/min. The maximum temperature was 350°C. The column was heated from 35°C to 290°C. The injector temperature was set at 300°C in a splitless mode. Results were recorded as percent of total peak areas. The mass spectrometer employed in the GC-MS analysis was a Polarization Q series mass selective detector in the electron impact (EI) ionization mode (70 eV). Using appropriate reference standards of FAMEs, Y_2+3 _was identified as a mixture of four FAs: palmitic acid (C16:0), isomers of C18:2 and C18:1 and stearic acid (C18:0) in the corresponding proportion 1:5:8:1.

### Treatment of cells with plant extracts and fatty acids

Plant extracts, fish oil, CLA, the FAs: palmitic, linoleic (cis-9, cis-12-octadecadienoic acid), oleic (cis-9-octadecenoic acid) and stearic acid and a mix of the four FAs (1:5:8:1 ratio) were all diluted in 100% EtOH and stored at -20°C. On day 3 after plating, SCp2 cells were treated with plant extracts or other FA compounds at different concentrations in DM supplemented with 1% FBS up to a final volume of 1 ml/well. Following treatment, cells were incubated at 37°C for different time points (24 or 48 hr) to assess cytotoxicity or for 30 min (short-term treatment) before ET treatment. For other experiments, media were supplemented with plant extracts or other FA compounds at different concentrations for 3 days as of the plating day (long-term treatment), and cells were treated with ET on day 3.

For Mode-K cells, on day 2 after plating, cultures were pretreated with Y_2+3 _or a synthetic FA mix at different concentrations in the absence of FBS up to a final volume of 1 ml/well. Following treatment, cells were incubated at 37°C for 8 or 12 hr before IL-1 treatment on day 3. Another method involved the cotreatment of Mode-K cells with Y_2+3 _or a synthetic FA mix and IL-1 on day 3 for 8 hr under similar conditions.

### Induction of inflammation

#### Endotoxin treatment of SCp2 cells

ET treatment was performed as previously described by Saliba *et al*. (2009) [24]. *Salmonella typhosa *ET (Sigma) was dissolved in DM, filter-sterilized using 0.2 μm non-pyrogenic sterile-R filter and stored at -20°C. On day 3 after plating, cells were treated with ET at 10 μg/ml and then incubated at 37°C for 9 hr.

#### Interleukin-1 treatment of Mode-K cells

IL-1 treatment was performed as previously described by Homaidan *et al*. (2003) [25]. IL-1α (US Biological, Swampscott, MA, USA) was dissolved in 1% bovine serum albumin (BSA; Invitrogen) and stored at -20°C. On day 3 after plating, cells were treated with IL-1 at 10 ng/ml and then incubated at 37°C for 6 or 8 hr.

### Trypan blue exclusion method

Twenty-four or 48 hr post plant treatment, viable and dead SCp2 cells were counted using the trypan blue exclusion method. It involves the trypsinization of the attached cells and washing them using the same treatment medium, which contains dead cells, to form a suspension of the total treated cells. An aliquot of 50 μl is taken from this suspension and mixed with an equal volume of trypan blue (Gibco). Dead cells stain blue, while viable cells appear bright. The percentage of viability is calculated relative to the control.

### Enzyme-linked immunosorbent assay (ELISA)

#### Sample collection

Media of SCp2 cells were sampled from triplicate wells 9 hr post ET treatment. Forty μl of complete protease inhibitors [one tablet dissolved in 2 ml of double distilled water (DDW); Roche Diagnostics GmbH, Mannheim, Germany] was added to each 1 ml of sample. Samples were stored at -80°C until the day of the assay.

#### Interleukin-6 assay

A two-site (sandwich) ELISA was performed for the quantitative determination of mouse IL-6 (mIL-6) present in SCp2 cell culture media, using mIL-6 ELISA immunoassay kit (BioSource International, Inc., Camarillo, CA, USA). The IL-6 assay was performed according to manufacturers' instructions. All standards and samples were run in duplicates on high-binding 96-well microtiter plates (Thermo Labsystems, Philadelphia, PA, USA). The optical density was measured at a wavelength of 450 nm by an ELISA microplate reader (Multiskan Ascent, Thermo Labsystems). Concentrations were calculated using the Ascent software and were expressed in pg/ml.

### Western blotting

Mode-K cells were washed twice with phosphate-buffered saline (PBS) and scraped in 2× electrophoresis sample buffer (SB) containing 0.25 M Tris-HCl (pH 6.8; Amersham Biosciences, San Diego, CA, USA), 4% w/v sodium dodecyl sulfate (SDS; Amersham Biosciences), 20% w/w glycerol (Amersham Biosciences), 0.1% bromophenol blue and 40 μl/ml protease inhibitor cocktail (Biomol, Plymouth Meeting, PA, USA). Samples were then collected in microfuge tubes, boiled for 5 min, centrifuged and the supernatant representing total soluble protein extract was collected and stored at -80°C.

Total protein extracts were run on a 12% SDS-polyacrylamide gel (BioRad, Hercules, CA, USA), and the gels were transferred to polyvinylidene difluoride (PVDF) membranes (Amersham Biosciences) overnight at 4°C. Following transfer, membranes were washed once with TPBS wash buffer (PBS containing 0.1% Tween 20) and then blocked in 5% non-fat dry milk for 2 hr at room temperature. Rabbit polyclonal COX-2 antibody (Cayman Chemical, Ann Arbor, MI, USA) was then added to the membranes and incubated for 2 hr at room temperature. Unbound antibodies were washed three times with TPBS. Horse-raddish peroxidase (HRP)-conjugated anti-rabbit IgG (Santa Cruz Biotechnology, Santa Cruz, CA, USA) was added at 1:5000 dilution for 1 hr at room temperature. Membranes were washed, incubated with luminol reagents (Santa Cruz Biotechnology) and directly exposed to autoradiography.

### Statistical analysis

Data were expressed as mean ± S.D. The effectiveness of plant treatments was analyzed by one-way analysis of variance (ANOVA). To check for treatments with similar effects, Student-Newman-Keuls (SNK) and Tukey HSD tests were performed. The effect of each treatment, if any, was then compared to the control using two-sample t-test or Dunnett t-tests for multiple comparisons. All statistical analyses were carried out using statistical program for social sciences (SPSS) 11.5, except for t-test, which was performed using Excel. Statistical probability of *P *< 0.05 was considered significant.

## Results

### Effect of *R. constantinopolitanus *extracts on endotoxin-induced interleukin-6 levels in SCp2 cells

**Figure 1:**
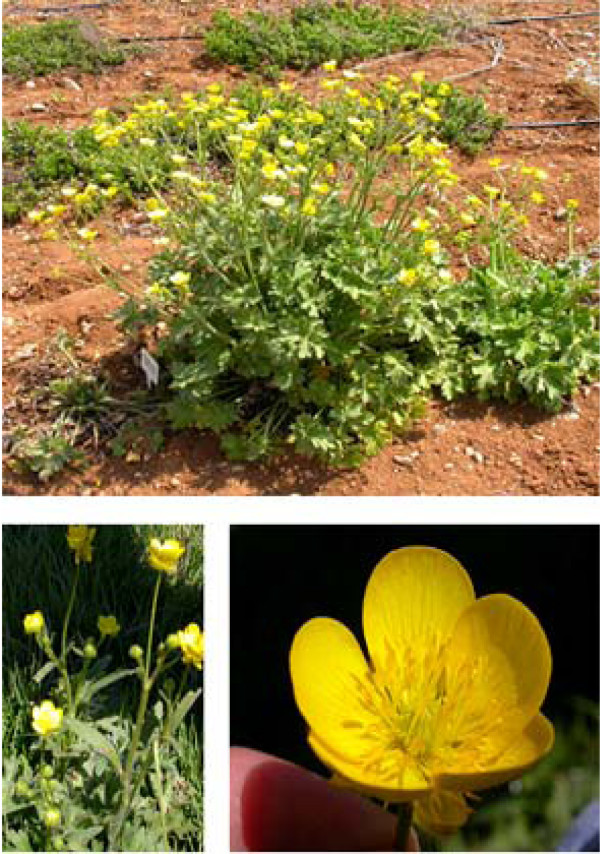
***Ranunculus constantinopolitanus***. *Ranunculus constantinopolitanus *(Arabic name: Hawdhan fa'ri) is an Eastern Mediterranean plant that grows in Aintab and extends from Hasbayya to Jazzin. Description: "Villous below, appressed-hairy above. Root-fibers descending directly from neck. Root-leaves triangular-ovate, ternate, with obovate, cut, and coarsely toothed lobes. Carpels large, ovate, striate, smooth, ending abruptly in a very short, hooked beak". Its flowering season falls between April and June [41]. The plant has been identified by Dr. Nada Sinno-Saoud, and a voucher specimen (voucher number: 72) was deposited at the Post Herbarium of the American University of Beirut, Beirut, Lebanon. Photos courtesy of Mr. Khaled Sleem (2004), Crop Production and Protection Department, Faculty of Agricultural and Food Sciences, American University of Beirut, Beirut, Lebanon.

**Figure 2:**
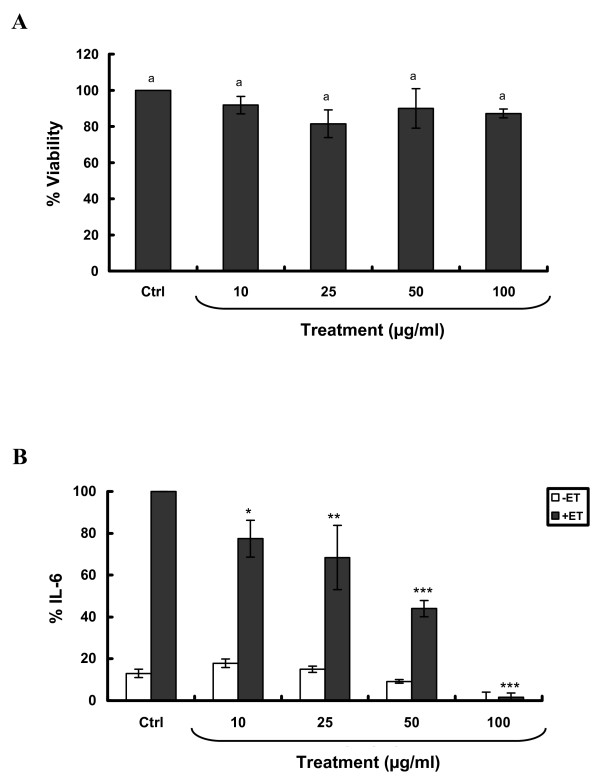
**Exposure of SCp2 cells to *R. constantinopolitanus *fraction I.2 at noncytotoxic concentrations reduces, in a concentration-dependent manner, ET-induced IL-6 levels**. SCp2 cells were treated on day 3 of culture with EtOH (Ctrl) or I.2 at different concentrations in DM supplemented with 1% FBS: **(A) **Twenty-four hr later, trypan blue assay was performed. **(B) **Thirty min later, cells were treated with ET at 10 μg/ml and their media collected 9 hr post-ET. The values depicted are the means (± S.D.) of a triplicate treatment. Means with the same letter are not significantly different. Statistical significance of the difference from Ctrl+ET is with *P < 0.05, **P < 0.01 or ***P < 0.001. (Ctrl, control; ET, endotoxin; IL-6, interleukin-6). -ET (white square); +ET (black square).

*Ranunculus constantinopolitanus *(Figure 1), known for its folk medicinal value, was subjected to chemical extraction and purification. The crude MeOH extract (I) from this plant was cytotoxic to SCp2 cells. Accordingly, I.2, the chloroform fraction derived from that extract, was tested for its biological activity on SCp2 cells. To determine the maximum concentration of I.2 that can be used without affecting cell viability or morphology, SCp2 cells were treated with I.2 at different concentrations (10, 25, 50 or 100 μg/ml) or EtOH, as a vehicle control, and viable cell counts were determined. None of the tested concentrations affected cell morphology 24 hr post-treatment (Data not shown), and cell viability for all concentrations did not vary significantly from the control treatment (Figure 2A). Thus, the ability of I.2 to inhibit ET-induced IL-6 was studied to evaluate its potential anti-inflammatory activities. SCp2 cells pretreated with I.2 at the different concentrations (10, 25, 50 or 100 μg/ml) showed, in a concentration-dependent manner, a significant reduction in IL-6 levels stimulated by ET (Figure 2B). Basal IL-6 levels were noted in cells pretreated with 100 μg/ml.

**Figure 3:**
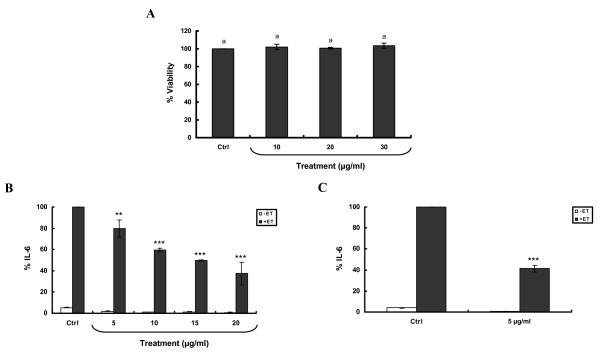
**Exposure of SCp2 cells to Y_2+3 _at noncytotoxic concentrations reduces, in a concentration-dependent manner, ET-induced IL-6 levels**. **(A) **SCp2 cells were treated on day 3 of culture with EtOH (Ctrl) or Y_2+3 _at different concentrations in DM supplemented with 1% FBS. Forty-eight hr later, trypan blue assay was performed. SCp2 cells were treated **(B) **on day 3 of culture 30 min before ET (short-term exposure) or **(C) **for 3 days (long-term exposure), as of day 1 of culture, with EtOH (Ctrl) or Y_2+3 _at different concentrations. Cells were treated on day 3 with ET at 10 μg/ml in DM supplemented with 1% FBS and their media collected 9 hr post-ET. The values depicted are the means (± S.D.) of a triplicate treatment. Means with the same letter are not significantly different. Statistical significance of the difference from Ctrl+ET is with **P < 0.01 or ***P < 0.001. (Ctrl, control; ET, endotoxin; IL-6, interleukin-6). -ET (white square); +ET (black square).

Treatment of SCp2 cells with Y_2+3 _at different concentrations (10, 20 or 30 μg/ml) or EtOH, as a vehicle control, did not alter cell morphology at any of the tested concentrations for up to 48 hr post-treatment (Data not shown). In addition, cell counts performed at the same time point showed that cell viability for each concentration was similar to that of the control treatment (Figure 3A). Consequently, Y_2+3 _was used at concentrations not exceeding 30 μg/ml in all subsequent experiments.

To check if the anti-inflammatory effect previously observed with I.2 was due to the Y_2+3 _subfraction, the ability of the latter to inhibit ET-induced IL-6 was evaluated. SCp2 cells were pretreated with different Y_2+3 _concentrations, not exceeding 30 μg/ml, using two modes of treatment. Short-term treatment at 5, 10, 15 or 20 μg/ml resulted in a concentration-dependent downregulation of ET-induced IL-6 levels (Figure 3B). This reduction was significant at all the tested concentrations and exceeded 50% inhibition at the highest Y_2+3 _concentration (20 μg/ml). Long-term treatment at 5 μg/ml significantly downregulated ET-induced IL-6 levels by more than 50% (Figure 3C).

### Potency of Y_2+3 _versus its presumed fatty acid components, fish oil or conjugated linoleic acid in regulating endotoxin-induced interleukin-6 levels in SCp2 cells

**Figure 4:**
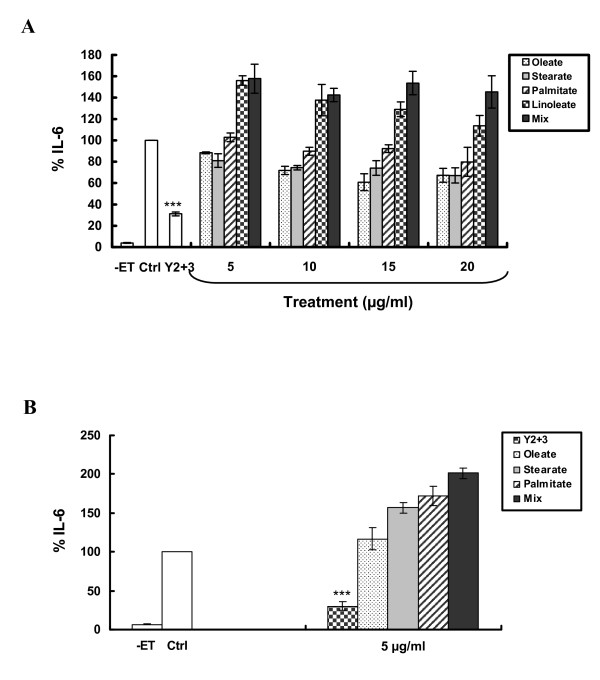
**Exposure of SCp2 cells to individual FA components of Y_2+3 _or a synthetic FA mix does not reduce ET-induced IL-6 levels**. SCp2 cells were treated **(A) **on day 3 of culture 30 min before ET (short-term exposure) or **(B) **for 3 days (long-term exposure), as of day 1 of culture, with EtOH (Ctrl), Y_2+3_^a^, palmitate, linoleate^b^, oleate, stearate or a synthetic FA mix at different concentrations. Cells were treated on day 3 with ET at 10 μg/ml in DM supplemented with 1% FBS and their media collected 9 hr post-ET. The values depicted are the means (± S.D.) of a triplicate treatment. Statistical significance of the difference from Ctrl+ET is with ***P < 0.001. (Ctrl, control; -ET, untreated cells; IL-6, interleukin-6). *^a^For short-term exposure **(A)**, cells were treated with Y_2+3 _at 10 μg/ml. ^b^Long-term exposure **(B) **to linoleate at 5 μg/ml was cytotoxic to cells*.

The effect of Y_2+3 _in inhibiting ET-induced IL-6 was compared to that of short- or long-term treatment with each of the individual FA constituents, i.e. palmitic and stearic acid and the two most common C18:2 and C18:1 isomers: linoleic (cis-9, cis-12-octadecadienoic acid) and oleic (cis-9-octadecenoic acid) acid, respectively, at concentrations similar to those previously used with Y_2+3_. It was noted that Y_2+3 _significantly reduced ET-induced IL-6 levels upon both short- and long-term treatment of SCp2 cells (Figure 4A and 4B). However, none of the individual FAs could reduce IL-6 levels at concentrations ranging from 5-20 μg/ml following short-term treatment (Figure 4A) or at a concentration of 5 μg/ml following long-term treatment (Figure 4B). Furthermore, neither short-term treatment at 5-20 μg/ml, nor long-term treatment at 5 μg/ml with a synthetic mix of palmitic, linoleic (cis-9, cis-12-octadecadienoic acid), oleic (cis-9-octadecenoic acid) and stearic acid in the same proportion as in Y_2+3 _could reduce ET-induced IL-6 levels (Figure 4A and 4B). In fact, in both modes of treatment the mix induced higher levels of IL-6 than those induced by ET alone, similarly for linoleate in short-term treatment and stearate and palmitate in long-term treatment. Noteworthy is that linoleate was cytotoxic to cells in long-term treatment mode.

**Figure 5:**
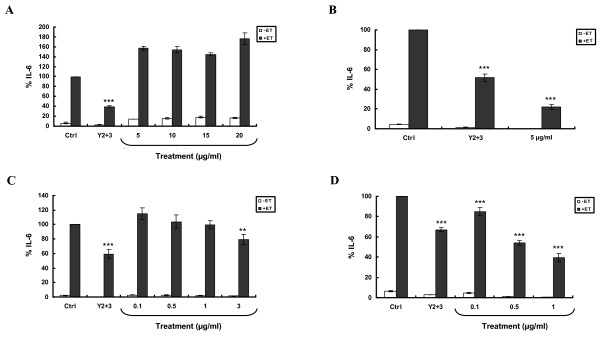
**Exposure of SCp2 cells to Y_2+3 _reduces ET-induced IL-6 levels more potently than fish oil and CLA**. SCp2 cells were treated **(A and C) **on day 3 of culture 30 min before ET (short-term exposure) or **(B and D) **for 3 days (long-term exposure), as of day 1 of culture, with EtOH (Ctrl), Y_2+3_^a^, fish oil **(A and B) **or CLA^b ^**(C and D) **at different concentrations. Cells were treated on day 3 with ET at 10 μg/ml in DM supplemented with 1% FBS and their media collected 9 hr post-ET. The values depicted are the means (± S.D.) of a triplicate treatment. Statistical significance of the difference from Ctrl+ET is with **P < 0.01 or ***P < 0.001. (Ctrl, control; ET, endotoxin; IL-6, interleukin-6). *^a^Cells were treated with Y_2+3 _at 5 or 10 μg/ml for long- **(B and D) **and short-term **(A and C) **exposure, respectively. ^b^Short- and long-term exposures to CLA at 5 and 3 μg/ml, respectively, were cytotoxic to cells*. -ET (white square); +ET (black square).

*n*-3 FAs and CLA are notoriously associated with anti-inflammatory properties and are available in many forms as over-the-counter supplements. The ability of fish oil, containing the *n*-3 FAs EPA and DHA (1:0.76 ratio), to reduce ET-induced IL-6 levels was compared to that noted with Y_2+3_. SCp2 cells were exposed to short- or long-term treatment with fish oil at concentrations similar to those used with Y_2+3_. In contrast to Y_2+3_, short-term fish oil treatment did not reduce IL-6 levels induced by ET, but rather enhanced such levels by at least 40% (Figure 5A). However, long-term treatment significantly reduced IL-6 to approximately 25% of the control levels (Figure 5B). Worth mentioning is the fact that fish oil reduced IL-6 levels more than Y_2+3 _at similar concentrations upon long-term treatment.

The ability of short- or long-term CLA treatment to reduce ET-induced IL-6 levels in SCp2 cells was also compared to that of Y_2+3_. At noncytotoxic concentrations (0.1, 0.5, 1 and 3 μg/ml), short-term CLA treatment did not reduce IL-6 levels, except for a marginal, but significant, reduction at 3 μg/ml (Figure 5C). This reduction was less than that observed with Y_2+3 _at 10 μg/ml. Following long-term treatment, CLA (0.1, 0.5 or 1 μg/ml) significantly reduced IL-6 levels more so than that noted for Y_2+3 _at the 0.5 and 1 μg/ml level (Figure 5D). Concentrations of CLA at 3 and 5 μg/ml were cytotoxic to cells upon long- and short-term treatment, respectively.

### Effect of Y_2+3 _on interleukin-1-induced cyclooxygenase-2 expression in Mode-K cells

**Figure 6:**
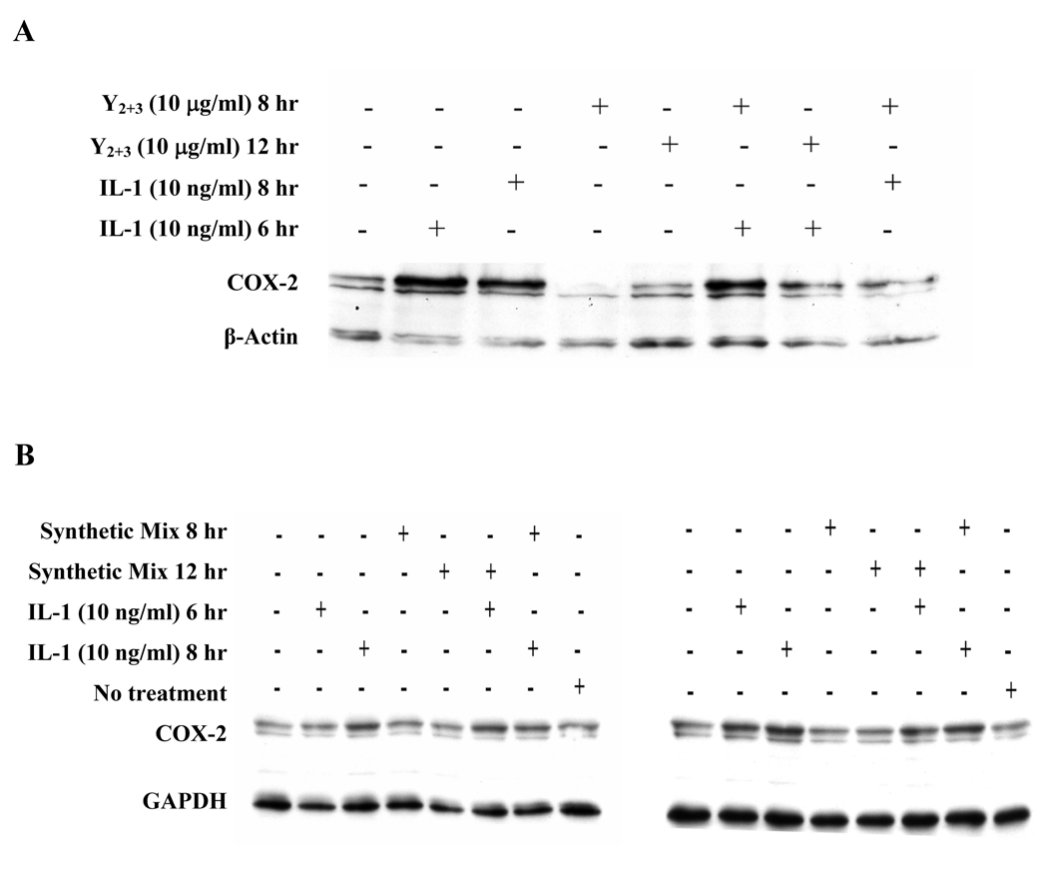
**Exposure of Mode-K cells to Y_2+3_, but not to a synthetic FA mix, reduces IL-1-induced COX-2 protein levels**. Mode-K cells were treated on day 2 of culture 8 or 12 hr before IL-1 (pretreatment) or on day 3 (cotreatment) with **(A) **Y_2+3 _at 10 μg/ml or **(B) **a synthetic FA mix at different concentrations (Left: 10 μg/ml; Right: 60 μg/ml) in FBS-free medium. Cells were treated on day 3 with IL-1 at 10 ng/ml in the absence of FBS and their proteins extracted 6 or 8 hr post-IL-1 for western blot analysis. β-actin or GAPDH was used to demonstrate equal loading. (COX-2, cyclooxygenase-2; IL-1, interleukin-1).

The effect of Y_2+3 _on COX-2 protein levels in Mode-K cells was assessed by pretreating the cells with Y_2+3 _at 10 μg/ml for 8 or 12 hr prior to 6-hr treatment with 10 ng/ml of IL-1 or cotreating cells with Y_2+3 _and IL-1 for 8 hr. Y_2+3 _caused a significant decrease in IL-1-induced COX-2 levels at 12-hr pretreatment as well as 8-hr cotreatment with IL-1 (Figure 6A). As with SCp2 cells, the effect of the synthetic mixture of FA components of Y_2+3 _on COX-2 protein levels in IL-1-treated Mode-K cells was tested. At 10 μg/ml, a similar concentration to Y_2+3_, the synthetic mix did not reverse the IL-1-induced COX-2 levels in Mode-K cells (Figure 6B, left panel). The synthetic mix failed to reverse these levels even at a higher concentration of 60 μg/ml (Figure 6B, right panel).

## Discussion

In this study, we report the existence of anti-inflammatory activities in *R. constantinopolitanus *(Arabic name: Hawdhan fa'ri), commonly used in Eastern Mediterranean folk medicine, using *in-vitro *models of inflammation. Previous work in our laboratory established an *in-vitro *model of inflammation [26]. In this model, treatment of mouse mammary cells with ET at 10 μg/ml, a noncytotoxic concentration, enhanced nuclear factor (NF)-κB DNA-binding activity, suppressed β-casein expression and upregulated pro-inflammatory mediators, including gelatinases (MMP-2 and MMP-9) and cytokines (IL-6 and TNF-α). Consequently, SCp2 mouse mammary epithelial cell strain was utilized to assess potential *in-vitro *anti-inflammatory activities in *R. constantinopolitanus*. In reviewing the literature, we highlighted the role phenols, flavonoids and terpenoids play in treating inflammation [27]. Accordingly, I.2, the chloroform fraction derived from the crude MeOH extract of the plant and presumably rich in these compounds, was assessed for its anti-inflammatory effects in ET-treated SCp2 cells. Due to its importance in the transition from the acute to the chronic phase of inflammation [28], IL-6 has been chosen as an inflammation marker to be monitored in our study. I.2 significantly reduced ET-induced IL-6 levels without altering cell viability or morphology. From there, I.2 was further fractionated and purified to yield a FA mix, Y_2+3_. Short- and long-term exposure to Y_2+3 _generated an IL-6 inhibitory profile similar to that of I.2 without causing cytotoxicity, but at relatively lower concentrations, indicating that the inhibitory effect noted in I.2 was possibly due to Y_2+3_.

Numerous reports have shown the occurrence of FAs in members of the Ranunculaceae family [29-31]. Chemical investigation demonstrated the presence of palmitic acid, C18:2 and C18:1 isomers and stearic acid (1:5:8:1 ratio) in Y_2+3_. In a similar study, these FAs, along with others, were reported to exist in *Ranunculus ternatus *[30], a Chinese medicinal plant used as an analgesic for headache and toothache and for treating congestion, corneal pterygium and malaria.

The comparison between the anti-inflammatory activity of Y_2+3 _and other commonly known biologically active FAs suggested that the higher potency of Y_2+3 _was not due to its presumed FAs, i.e. palmitic and stearic acid and at least not the two most common isomers of C18:2 and C18:1: linoleic and oleic acid, respectively. Even when tested at concentrations exceeding the ones normally present in Y_2+3 _mix, none of the FAs reduced ET-induced IL-6 levels. Furthermore, the synthetic FA mix, containing these FAs in the same ratio as in Y_2+3_, did not reduce IL-6 levels in SCp2 cells. Y_2+3 _has demonstrated comparable anti-inflammatory effects in IL-1-stimulated mouse intestinal epithelial Mode-K cells. Y_2+3 _effectively downregulated COX-2 expression in these cells, an activity not noted in its synthetic FA mix. Nevertheless, the above observations do not exclude potential combinatorial effects for these FAs. This suggestion is supported by a study that demonstrated antifertility activities in *Azadirachta indica *seed extract and in one of its fractions (containing palmitate, linoleate, oleate and stearate, in addition to methyl palmitate and methyl oleate), which were reduced upon further fractionation. In the same study, regrouping the obtained subfractions to reconstitute the original mixture in a similar proportion did not regenerate the original biological activity [32]. This was attributed to the synergism of the constituents of the mixture as they existed in the seed. Although this could be the case in our study, however, our results can be explained also by possible occurrence of CLA and/or its precursor vaccenic acid as the C18:2 and C18:1 isomers, respectively. Knowing that the anti-inflammatory role of CLA, unlike oleate, palmitate and stearate, was highlighted by different studies [9,14], we opted to investigate the effect of CLA on IL-6 production by SCp2 cells.

In addition, various reports demonstrated the efficacy of CLA, EPA and DHA in reversing inflammation [2,33-36]. Our comparative studies denote similar or more potent effects for Y_2+3 _in SCp2 cell culture model that could be noticed within a shorter exposure time or with less cytotoxic effects. Compared to fish oil, Y_2+3 _was able to reduce IL-6 levels within a shorter period of time; however, at longer exposures, fish oil was more potent than Y_2+3_. Similarly, CLA was less effective than Y_2+3_, even at the maximum noncytotoxic concentration for short durations, but exhibited more potent effect after long-term exposure. This suggests the presence of biologically active CLA isomer(s) in Y_2+3 _that could be synergized by the other FA constituents of Y_2+3_. Worth noting is the presence of a marketable formula of CLA that contains, in addition, palmitic, linoleic, oleic and stearic acid (Tonalin CLA, WeightLossGuide, Sarasota, FL, USA).

Studies have confirmed that dietary PUFA supplementation is capable of ameliorating IBD [37,38]. For instance, using a pig model of dextran sodium sulfate (DSS)-induced colitis, both *n*-3 PUFA- and CLA-rich diets were shown to exert anti-inflammatory effects [39]. In a related preliminary study, Y_2+3 _synergized the anti-inflammatory activities in salograviolide A (SA), a sesquiterpene lactone purified from *Centaurea ainetensis *[24,40], whereby intraperitoneal (i.p.) administration of Y_2+3_:SA mix reduced pro-inflammatory cytokine levels in colons of iodoacetamide-induced ulcerative colitis (UC) rats (Data not shown).

## Conclusion

Y_2+3_, the naturally occurring FA combination derived from *R. constantinopolitanus*, reduces ET-induced IL-6 levels and IL-1-induced COX-2 expression *in-vitro *more efficiently than the highly reputable CLA and fish oil and within a shorter period of time. Our preliminary studies demonstrate similar effects for Y_2+3 _in an *in-vivo *model of IBD, making it a promising plant-derived anti-inflammatory FA mix. The mechanism of action of this mixture and the nature of its C18:1 and C18:2 isomers have not been fully elucidated. Finally, a better understanding of the specific cellular targets of Y_2+3 _could prove invaluable in determining the role played by FAs in inflammation prevention and resolution.

## Competing interests

The authors declare that they have no competing interests.

## Authors' contributions

NAS, RAE and RGS carried out the isolation and identification of plant extracts. RST, FRH, SFF and JAA performed *in-vitro *studies on SCp2 (RST and SFF) and Mode-K cells (FRH and JAA) and outlined mechanisms of action. RST, FRH and NAS were the primary investigators who designed the experiments. SFF carried out the statistical analysis and prepared the manuscript. All authors read and approved the final manuscript.

## Pre-publication history

The pre-publication history for this paper can be accessed here:

http://www.biomedcentral.com/1472-6882/9/44/prepub
